# Concerns and Anxieties of Patients During Hospitalization for COVID-19

**DOI:** 10.7759/cureus.18202

**Published:** 2021-09-22

**Authors:** Ken Goda, Tsuneaki Kenzaka, Shinsuke Yahata, Ayako Kumabe, Masahiro Katsurada, Hogara Nishisaki

**Affiliations:** 1 Division of Community Medicine and Career Development, Kobe University Graduate School of Medicine, Kobe, JPN; 2 Department of Internal Medicine, Hyogo Prefectural Tamba Medical Center, Tanba, JPN; 3 Division of Community Medicine and Medical Education, Kobe University Graduate School of Medicine, Kobe, JPN

**Keywords:** covid-19, stigma, mental health, anxiety, health care providers

## Abstract

Objective

The objective of this study was to evaluate the concerns and anxieties of hospitalized coronavirus disease (COVID-19) patients.

Methods

A questionnaire was distributed to patients on discharge. The responses were analyzed once they were returned by mail.

Results

Responses were received from 27 of 39 patients (average age, 50 ± 17 years). Among the participants, 16 were male (59.3%), 19 were symptomatic (70.4%), and two required a ventilator (7.4%). Anxiety about symptom exacerbation was reported by 36.0% of participants. Quarantine-associated stress was experienced by 60.0% of participants, and 72.0% of participants supported the change in federal policy that allows asymptomatic patients and patients with mild conditions to isolate themselves at a hotel or their home. Following discharge, 44.0% of participants experienced anxieties regarding their lives after discharge, and 56.0% were anxious regarding discrimination and rumors. During hospitalization, 68.0% of participants re-evaluated their attitude toward health, 44.0% regretted the preventative measures they took before contracting COVID-19, and 44.0% felt guilty for becoming infected.

Conclusions

Participants experienced various kinds of stress related to hospitalization for COVID-19. There was a trend of people being more concerned about their relationships than their own health. Therefore, as asymptomatic participants and participants with mild symptoms also experienced psychological stresses, it is also necessary to consider the psychological and social effects of the disease.

## Introduction

The global pandemic caused by the novel coronavirus disease (COVID-19) is causing major challenges in the field of medicine. In addition to the major impact of COVID-19, several indirect impacts such as social distancing and economic challenges continue to persist and burden people’s daily lives. Furthermore, psychological after-effects [1] such as depressive symptoms, anxiety disorders, and post-traumatic stress disorders (PTSD) [2] have been reported as mental health consequences brought on by the pandemic.

Notably, the infection spread by asymptomatic patients is considered a concern [3], whereby many patients are forced into hospitalization even if they have mild symptoms. Although not limited to COVID-19, the changes in the environment caused by hospitalization are known to deteriorate a patient’s psychological state [4]. In addition, social issues such as discrimination increased globally through the COVID-19 pandemic, demanding media and government attention [5].

Although the pathology and treatment approaches for COVID-19 are now well known, there are currently few reports on the living conditions and mental status of COVID-19 patients during hospitalization. In this study, we aimed to evaluate the concerns and feelings of COVID-19 patients who were hospitalized.

## Materials and methods

Study design

This prospective observational survey was approved by the ethics committee of Hyogo Prefectural Tamba Medical Center (approval number: Tan-I number 1050). Since the questionnaire indicated that the responses would be used for research, the ethics committee waived the need for obtaining informed consent from the participants. The objectives of the study and use of data were clearly stated in the questionnaire distributed to the participants, and written consent was obtained for the publication of the results.

The study commenced during the early stage of the outbreak, when COVID-19 was just becoming prevalent in Japan. No appropriate previous studies were found and thus, we could not calculate the sample size needed to achieve statistical significance; thus the present study’s sample size may prove inadequate. However, as a pilot study, all patients admitted to our hospital with COVID-19 were included.

Participants

The participants were all adult patients (≧18 years old) admitted to Hyogo Prefectural Tamba Medical Center for COVID-19 from March 1st to September 30th, 2020.

Administration of the questionnaire

The questionnaire was distributed to patients at the time of their discharge to inquire about their anxieties and concerns during hospitalization. Patients took the questionnaire home and mailed it back within a month of their discharge. The survey questionnaire provided information on individual characteristics, along with their thoughts and feelings during hospitalization. Taking into consideration the timing and various waves of the COVID-19 epidemic in Japan, we defined the first wave as March to June 2020 and the second wave as July to September 2020.

Details of the questionnaire

Since there were no appropriate or relevant previous studies found, the questions for this study were developed and thoroughly examined by the co-authors before deciding the finalized contents.

As basic attributes, questions included the following: age, sex, address, occupation, household members, the reason for taking a polymerase chain reaction (PCR) test, symptomatic or asymptomatic at the time of hospitalization, specific symptoms, whether treated for COVID-19, specific treatment, duration of hospitalization, duration of the hotel stay for quarantine, duration in which the normal lifestyle was interrupted such as missing work before hospitalization, and time it took until the resumption of normal lifestyle/work after discharge from hospitalization.

The response items for questions regarding symptoms at the time of admission included fever, cough, sputum, sore throat, runny nose, wheezing, difficulty breathing, chest pain, muscle pain, joint pain, headache, disturbance in consciousness, fatigue, abdominal pain, nausea, vomiting, diarrhea, taste disorder, and loss of smell.

Data analysis

We performed descriptive statistics regarding questions about basic characteristics of participants along with impressions and mindsets during their hospitalization for COVID-19. Stata MP version 15 (StataCorp, College Station, TX, USA) was used for the analysis.

## Results

We received the questionnaire from 27 out of 39 patients who were hospitalized at Hyogo Prefectural Tamba Medical Center for COVID-19 (response rate of 69.2%). Table 1 shows the basic characteristics of these 27 participants (missing responses were excluded from the calculations). The mean age ± standard deviation was 50.0±17.8 years. Sixteen out of 27 were male (59.3%). While two participants (7.7%) were residents of Tamba City or Tambasasayama City, which constitutes the medical district where our hospital is located, the majority of the study participants were residents of other medical districts. As the reason for doing the PCR test, 11 participants responded that they were symptomatic (42.3%), two responded potential exposure to a cluster (7.7%), and 11 responded close contact (42.3%).

**Table 1:** Participant characteristics SD=standard deviation; PCR=polymerase chain reaction

	All		1st wave		2nd wave	
	N	n (%)	N	n (%)	N	n (%)
Sex (male)	27	16 (59.3)	16	11 (68.8)	11	5 (45.5)
Mean age (SD)	27	50.0 (17.8)	16	48.4 (15.2)	11	52.5 (21.6)
City	26		15		11	
Tamba		0 (0.0)		0 (0.0)		0 (0.0)
Tambasasayama		2 (7.7)		0 (0.0)		2 (18.2)
Other		24 (92.3)		15 (100.0)		9 (81.8)
Occupation (health care related)	24	2 (8.3)	14	2 (14.3)	10	0 (0.0)
Living alone	25	6 (24.0)	15	2 (13.3)	10	2 (13.3)
Reason for PCR test	26		15		11	
No contact but symptomatic		11 (42.3)		5 (33.3)		6 (54.6)
Exposure to a cluster		2 (7.7)		1 (6.7)		1 (9.1)
Close contact		11 (42.3)		9 (60.0)		2 (18.2)
Other		2 (7.7)		0 (0.0)		2 (18.2)
Treatments provided	27	7 (25.9)	16	4 (25.0)	11	3 (27.3)
Antipyretic and analgesic drugs	27	7 (25.9)	16	4 (25.0)	11	3 (27.3)
Oral medication for COVID-19	27	0 (0.0)	16	0 (0.0)	11	0 (0.0)
Inhaler for COVID-19	27	0 (0.0)	16	0 (0.0)	11	0 (0.0)
Oxygen therapy	27	1 (3.7)	16	1 (6.3)	11	0 (0.0)
Ventilator	27	2 (7.4)	16	2 (12.5)	11	0 (0.0)
Duration of hospitalization [mean (SD)]	27	10.4 (6.8)	16	11.8 (8.3)	11	8.3 (2.8)
Duration of hotel quarantine [mean (SD)]	27	5.4 (7.6)	16	8.9 (8.2)	11	0.2 (0.6)
Duration in which normal lifestyle was interrupted such as missing work before hospitalization [mean (SD)]	24	5.8 (7.1)	14	6.1 (5.8)	10	5.4 (9.0)
Time it took until normal lifestyle/work was resumed (upon returning home after discharge from the medical center or quarantine hotel) [mean (SD)]	25	9.4 (10.4)	15	10.9 (12.5)	10	7.2 (6.0)

Table 2 shows a breakdown of symptoms at the time of hospitalization. Eight participants were asymptomatic and 19 participants were symptomatic (70.4%). In the order of the higher prevalence, symptoms were fever (n=12, 44.4%), fatigue (n=10, 37.0%), cough (n=9, 33.3%) headache (n=7, 25.9%), taste disorder (n=7, 25.9%), loss of smell (n=7, 25.9%), and sputum (n=6, 22.2%). Two participants were in serious condition and required invasive use of a ventilator (7.4％) while one participant required oxygen therapy. The majority of the participants experienced relatively mild symptoms or were asymptomatic (88.8%). The mean duration of hospitalization was 10.4 ± 6.8 days. The duration before hospitalization, when participants were unable to live their normal routine and missed work, was 5.8 ± 7.1 days. It took 9.4 ± 10.4 days after leaving the medical center or a quarantined hotel for participants to return to normal life and work.

**Table 2:** Symptoms at the time of hospitalization

	All (N=27)	1st wave (N=16)	2nd wave (N=11)
	n (%)	n (%)	n (%)
Symptomatic at the time of hospitalization	19 (70.4)	12 (75.0)	7 (63.6)
Fever	12 (44.4)	8 (50.0)	4 (36.4)
Cough	9 (33.3)	5 (31.3)	4 (36.4)
Sputum	6 (22.2)	2 (12.5)	4 (36.4)
Sore throat	4 (14.8)	2 (12.5)	2 (18.2)
Runny nose	1 (3.7)	0 (0.0)	1 (9.1)
Wheezing	1 (3.7)	1 (6.3)	0 (0.0)
Difficulty breathing	4 (14.8)	4 (25.0)	0 (0.0)
Chest pain	1 (3.7)	1 (6.3)	0 (0.0)
Muscle pain	1 (3.7)	1 (6.3)	0 (0.0)
Joint pain	2 (7.4)	1 (6.3)	1 (9.1)
Headache	7 (25.9)	3 (18.8)	4 (36.4)
Disturbance in consciousness	1 (3.7)	1 (6.3)	0 (0.0)
Fatigue	10 (37.0)	8 (50.0)	2 (18.2)
Abdominal pain	0 (0.0)	0 (0.0)	0 (0.0)
Nausea	4 (14.8)	3 (18.8)	1 (9.1)
Vomiting	0 (0.0)	0 (0.0)	0 (0.0)
Diarrhea	5 (18.5)	3 (18.8)	2 (18.2)
Taste disorder	7 (25.9)	4 (25.0)	3 (27.3)
Loss of smell	7 (25.9)	4 (25.0)	3 (27.3)

Concerns on being hospitalized

Table 3 shows the results of the concerns 25 participants had about their hospitalization. Since most participants experienced mild symptoms or were asymptomatic (88.8%), almost 70% found their hospitalization stay boring. However, the percentage of participants who felt this way was lower in the second wave (85.7% in the first wave vs. 45.5% in the second wave). The participants were highly satisfied with the hospital room, and 90% found the explanations and responses provided by medical care staff were adequate. About two-thirds of participants were satisfied with meals and night-time sleep. On the other hand, more than 80% responded that there was a lack of exercise.

**Table 3:** Concerns on being hospitalized

Concerns on being hospitalized		Strongly agree	Agree	Neither agree nor disagree	Disagree	Strongly disagree
	N	n (%)	n (%)	n (%)	n (%)	n (%)
Was being hospitalized boring?						
All	25	11 (44.0)	6 (24.0)	3 (12.0)	1 (4.0)	4 (16.0)
1st wave	14	8 (57.1)	4 (28.6)	0 (0.0)	1 (7.1)	1 (7.1)
2nd wave	11	3 (27.3)	2 (18.2)	3 (27.3)	0 (0.0)	3 (27.3)
Was the hospital room comfortable?						
All	25	16 (64.0)	8 (32.0)	1 (4.0)	0 (0.0)	0 (0.0)
1st wave	14	8 (57.1)	5 (35.7)	1 (7.1)	0 (0.0)	0 (0.0)
2nd wave	11	8 (72.7)	3 (27.3)	0 (0.0)	0 (0.0)	0 (0.0)
Were explanations and responses provided by health care providers adequate?						
All	25	18 (72.0)	4 (16.0)	2 (8.0)	1 (4.0)	0 (0.0)
1st wave	14	10 (71.4)	2 (14.3)	2 (14.3)	0 (0.0)	0 (0.0)
2nd wave	11	8 (72.7)	2 (18.2)	0 (0.0)	1 (9.1)	0 (0.0)
Were meals enjoyable?						
All	25	7 (28.0)	8 (32.0)	5 (20.0)	4 (16.0)	1 (4.0)
1st wave	14	5 (35.7)	4 (28.6)	2 (14.3)	3 (21.4)	0 (0.0)
2nd wave	11	2 (18.2)	4 (36.4)	3 (27.3)	1 (9.1)	1 (9.1)
Were you able to sleep well at night?						
All	25	11 (44.0)	6 (24.0)	5 (20.0)	2 (8.0)	1 (4.0)
1st wave	14	7 (50.0)	4 (28.6)	1 (7.1)	1 (7.1)	1 (7.1)
2nd wave	11	4 (36.4)	2 (18.2)	4 (36.4)	1 (9.1)	0 (0.0)
Were you able to get enough exercise?						
All	25	0 (0.0)	1 (4.0)	3 (12.0)	8 (32.0)	13 (52.0)
1st wave	14	0 (0.0)	0 (0.0)	2 (14.3)	4 (28.6)	8 (57.1)
2nd wave	11	0 (0.0)	1 (9.1)	1 (9.1)	4 (36.4)	5 (45.5)

Feelings concerning hospitalization

Table 4 shows the results of the feelings 25 participants had concerning their hospitalization. As most participants experienced only mild symptoms or were asymptomatic, 72% felt physically well. Meanwhile, 36% were anxious about their symptoms getting worse. The percentage of participants feeling anxious about their symptoms increased, from 28.5% in the first wave to 45.5% in the second wave, and 60% experienced stress from being quarantined.

**Table 4:** Feelings concerning hospitalization

Feelings concerning hospitalization		Strongly agree	Agree	Neither agree nor disagree	Disagree	Strongly disagree	No answer
	N	n (%)	n (%)	n (%)	n (%)	n (%)	
Did you feel physically well?							
All	25	13 (52.0)	5 (20.0)	3 (12.0)	2 (8.0)	1 (4.0)	1 (4.0)
1st wave	14	6 (42.9)	4 (28.6)	0 (0.0)	2 (14.3)	1 (7.1)	1 (7.1)
2nd wave	11	7 (63.6)	1 (9.1)	3 (27.3)	0 (0.0)	0 (0.0)	0 (0.0)
Did you feel anxious about symptoms getting worse?							
All	25	4 (16.0)	5 (20.0)	2 (8.0)	5 (20.0)	8 (32.0)	1（4.0）
1st wave	14	1 (7.1)	3 (21.4)	2 (14.3)	3 (21.4)	5 (35.7)	0 (0.0)
2nd wave	11	3 (27.3)	2 (18.2)	0 (0.0)	2 (18.2)	3 (27.3)	1（9.1）
Did you feel stressed about being quarantined at the medical center?							
All	25	7 (28.0)	8 (32.0)	4 (16.0)	1 (4.0)	5 (20.0)	0（0.0）
1st wave	14	6 (42.9)	4 (28.6)	2 (14.3)	1 (7.1)	1 (7.1)	0（0.0）
2nd wave	11	1 (9.1)	4 (36.4)	2 (18.2)	0 (0.0)	4 (36.4)	0（0.0）
Were you afraid of medical care staff wearing full PPE?							
All	25	3 (12.0)	2 (8.0)	2 (8.0)	1 (4.0)	17 (68.0)	0（0.0）
1st wave	14	2 (14.3)	1 (7.1)	1 (7.1)	0（0.0）	10 (71.4)	0（0.0）
2nd wave	11	1 (9.1)	1 (9.1)	1 (9.1)	1 (9.1)	7 (63.6)	0（0.0）
Did you feel anxious about difficulty getting information from outside of the medical center?							
All	25	3 (12.0)	3 (12.0)	2 (8.0)	5 (20.0)	11 (44.0)	1（4.0）
1st wave	14	2 (14.3)	2 (14.3)	2 (14.3)	3 (21.4)	5 (35.7)	0（0.0）
2nd wave	11	1 (9.1)	1 (9.1)	0 (0.0)	2 (18.2)	6 (54.5)	1 (9.1)
Did you find it difficult not being able to see your family?							
All	25	8 (32.0)	3 (12.0)	4 (16.0)	2 (8.0)	7 (28.0)	1（4.0）
1st wave	14	6 (42.9)	0 (0.0)	3 (21.4)	1 (7.1)	3 (21.4)	1 (7.1)
2nd wave	11	2 (18.2)	3 (27.3)	1 (9.1)	1 (9.1)	4 (36.4)	0（0.0）
Did you contact your family during your hospitalization? (yes)							
All	25	24 (96.0)					
1st wave	14	14 (100.0)					
2nd wave	11	10 (90.9)					
Did you feel that it would have been better to isolate yourself at home while you were being hospitalized?							
All	25	8 (32.0)	6 (24.0)	3 (12.0)	1 (4.0)	7 (28.0)	0（0.0）
1st wave	14	6 (42.9)	2 (14.3)	2 (14.3)	0（0.0）	4 (28.6)	0（0.0）
2nd wave	11	2 (18.2)	4 (36.4)	1 (9.1)	1 (9.1)	3 (27.3)	0（0.0）
Do you think it is a good idea that asymptomatic patients and patients with mild symptoms are now allowed to isolate at a hotel or at home?							
All	25	7 (28.0)	11 (44.0)	2 (8.0)	2 (8.0)	2 (8.0)	1（4.0）
1st wave	14	5 (35.7)	7 (50.0)	1 (7.1)	0 (0.0)	1 (7.1)	0 (0.0)
2nd wave	11	2 (18.2)	4 (36.4)	1 (9.1)	2 (18.2)	1 （9.1)	1 （9.1)
Did you feel anxious about your life after being discharged?							
All	25	6 (24.0)	5 (20.0)	2 (8.0)	5 (20.0)	7 (28.0)	0（0.0）
1st wave	14	2 (14.3)	4 (28.6)	1 (7.1)	3 (21.4)	4 (28.6)	0（0.0）
2nd wave	11	4 (36.4)	1 (9.1)	1 (9.1)	2 (18.2)	3 (27.3)	0（0.0）
Did you feel anxious if your family, colleagues, and friends would treat you differently after hospitalization?							
All	25	5 (20.0)	7 (28.0)	3 (12.0)	3 (12.0)	6 (24.0)	1（4.0）
1st wave	14	3 (21.4)	2 (14.3)	3 (21.4)	2 (14.3)	4 (28.6)	0 (0.0)
2nd wave	11	2 (18.1)	5 (45.5)	0 (0.0)	1 (9.1)	2 (18.1)	1（9.1)
Did you feel anxious if "you might have infected" your family, colleagues, and friends?							
All	25	6 (26.1)	6 (26.1)	3 (13.0)	3 (13.0)	5 (21.7)	2（8.0）
1st wave	14	1 (7.1)	5 (35.7)	2 (14.3)	2 (14.3)	3 (21.4)	1 (7.1)
2nd wave	11	5 (45.5)	1 (9.1)	1 (9.1)	1 (9.1)	2 (18.2)	1 (9.1)
Did you feel anxious about the possibilities of discrimination and rumors after being discharged?							
All	25	7 (28.0)	7 (28.0)	1 (4.0)	1 (4.0)	8 (32.0)	1 (4.0)
1st wave	14	4 (28.6)	3 (21.4)	1 (7.1)	0 (0.0)	6 (42.9)	0 (0.0)
2nd wave	11	3 (27.3)	4 (36.4)	0 (0.0)	1 (9.1)	2 (18.2)	1 (9.1)
Did you re-evaluate and become more aware of your view on your own health during the hospitalization?							
All	25	10 (40.0)	7 (28.0)	3 (12.0)	3 (12.0)	1 (4.0)	1 (4.0)
1st wave	14	2 (14.3)	6 (42.9)	3 (21.4)	3 (21.4)	0 (0.0)	0 (0.0)
2nd wave	11	8 (72.7)	1 (9.1)	0 (0.0)	0 (0.0)	1 (9.1)	1 (9.1)
Did you regret your infection control measures?							
All	25	7 (28.0)	4 (16.0)	7 (28.0)	3 (12.0)	3 (12.0)	1（4.0）
1st wave	14	3 (21.4)	3 (21.4)	6 (42.9)	1 (7.1)	1 (7.1)	0（0.0）
2nd wave	11	4 (36.4)	1 (9.1)	1 (9.1)	2 (18.2)	2 (18.2)	1 (9.1)
Did you feel guilty about becoming infected?							
All	25	7 (28.0)	4 (16.0)	2 (8.0)	6 (24.0)	5 (20.0)	1（4.0）
1st wave	14	3 (21.4)	2 (14.3)	1 (7.1)	3 (21.4)	5 (35.7)	0（0.0）
2nd wave	11	4 (36.4)	2 (18.2)	1 (9.1)	3 (27.3)	0（0.0）	1（9.1）

In terms of medical care staff wearing the full PPE, 72% responded that they had no issue. About 65% felt no anxiety over the lack of information from outside of the medical center.

Almost all participants (96.0%) were in contact with their families during the hospitalization, and about half of the participants found it difficult not being able to see their families.

Since most participants experienced mild symptoms or were asymptomatic, half of them felt that they could have isolated themselves at home instead of being hospitalized. About 70% felt that it was a good idea to change the federal policy from hospitalization of all positive participants to hotel quarantine and home isolation for asymptomatic participants and those with mild symptoms.

About half of the participants were anxious about life after getting discharged, wherein about 50% were concerned if family, colleagues, and friends would treat them differently. Moreover, about 60% were concerned about facing possible discrimination and rumors after getting discharged, and almost 70% said that they re-evaluated their views on their own health during the hospitalization. Just over 40% regretted their own infection control measures, and also felt guilty about getting infected.

## Discussion

In this study, 24 out of 27 participants (88.8%) experienced mild symptoms or were asymptomatic and did not require oxygen therapy. Nevertheless, participants who were hospitalized for COVID-19 experienced various forms of stress regarding hospitalization. Specifically, there was a tendency to worry about inter-personal relationships more than their own physical conditions. In this regard, it needs to be acknowledged that those who are asymptomatic or only experiencing mild symptoms also experience psychological stress, and therefore, measures to alleviate such stress are necessary.

As far as the experience during hospitalization is concerned, many found it boring. This is likely because many participants were forced into hospitalization despite being asymptomatic or not needing hospitalization because of concern for asymptomatic or pre-symptomatic transmissions [3]. Most participants were satisfied with the hospital room setting and explanations provided by health care staff, but there were restrictions on meals and sleep: specifically, 84% felt they had restrictions on exercise. These results indicate that there were dramatic changes in the environment due to hospitalization. Although the participants felt well, they were still confined to their room, which led to a lack of exercise.

While most of the participants were in contact with their families and could express their feelings during hospitalization, it also seemed that there was little anxiety and stress about keeping in contact with their families. Moreover, anxiety regarding receiving information and news during the hospital stay also seemed minimal. There were concerns about "participant psychological stress" when exposed to health care workers wearing full PPE, however, participants were not particularly stressed by the extraordinary appearance of the attending medical staff.

On the other hand, similar to restricted exercise, 60% of participants felt stressed about quarantine. The mean duration of hospitalization was 10.4 ± 6.8 days, mostly 10 days to two weeks. However, this may be long enough to cause stress for asymptomatic individuals or those with only mild symptoms. The present policy is that those with no symptoms or only mild symptoms, without any underlying conditions that could cause severe infection, are to isolate at a hotel or at home. For those patients, the stress of isolation is minimized by keeping a line of conversation open with staff by using a remote camera or similar device.

Instead of worrying about their physical conditions, such as worsening of symptoms, many participants were more concerned about the relationships--"how family, colleagues, and friends might react after the discharge," "they might have infected someone," or discrimination and rumors after the discharge.

COVID-19 is known to be a risk factor for mental health problems [1]. Unpredictable and uncertain situations, social isolation through lockdowns and physical distancing, reduced income and loneliness, and reduced activities and restricted movements are said to increase drinking and gambling and lead to reduced family and social support for seniors [1]. In addition, depressive symptoms, anxiety disorders, and PTSD have been reported as mental health complications or consequences of COVID-19 [2]. On the other hand, not limited to COVID-19, changes in the environment caused by hospitalization are known to worsen the patients' psychological status [4]. As the present survey showed that participants were dealing with various anxieties, it is important to not only focus on the physical harm caused by COVID-19, but also ensure that patients are in a sound mental state during their hospitalization, and provide the necessary subsequent mental support from clinical psychologists or other mental health professionals.

As for discrimination, a global survey conducted on 1,904 Chinese people living in 70 countries in February 2020 showed that 25.11% of respondents experienced discrimination, such as unreasonable job dismissals, denial of residential rentals, and abuse in public spaces [5]. Additionally, in a survey conducted on 17,846 people living in 31 provinces in mainland China (including direct jurisdictions, special administrative regions, and cities), about 90% of respondents said that if they were to discover someone from Hubei province in their own region, they would report it to the authorities, 50.58% said that they would avoid people from Hubei province, and 16.94% said that they would actively drive out people from Hubei province [5].

Moreover, social issues such as discrimination arose globally through the COVID-19 pandemic and made severe impacts, alerting governments and media outlets [5]. In addition, COVID-19 is a risk factor for mental health problems, accompanied by environmental changes caused by hospitalization, discrimination, and rumors surrounding the disease. As such, the adverse effects and psychological impacts of COVID-19 represent a growing concern. However, this study showed that hospitalization for COVID-19 provided patients with an opportunity to re-evaluate their own health.

Limitations

This study has some limitations. First, the present study was conducted at one facility since the beginning of the COVID-19 pandemic. Second, since the number of cases was low in the early stage, we suggest caution in generalizing the present results.

## Conclusions

Patients hospitalized for treatment of COVID-19 experienced various kinds of stress. Being hospitalized in an area outside of their own town or city and having their movements restricted had an especially notable impact on their mental well-being. Psychologically, there was a tendency to worry about relationships with others instead of their own physical condition. Therefore, we need to recognize that even those who are asymptomatic or have mild symptoms experience psychological stress and take steps to reduce that stress. As the pandemic evolves, apart from the treatment of the disease itself, it will be essential to take into account the psychological and social effects of contracting the disease.

## Appendices

The following questions were asked regarding the concerns of being hospitalized for COVID-19. The answers were provided using a five-point Likert scale (strongly agree, agree, neither agree nor disagree, disagree, and strongly disagree).

1. Was being hospitalized boring?

2. Was the hospital room comfortable?

3. Were explanations and responses provided by health care providers adequate?

4. Were meals enjoyable?

5. Were you able to sleep well at night?

6. Were you able to get enough exercise?

The following questions were asked to assess the feelings of participants during their hospitalization for COVID-19. The answers were also provided using a five-point Likert scale, excluding some questions.

1. Did you feel physically well?

2. Did you feel anxious about symptoms getting worse?

3. Did you feel stressed about being quarantined at the medical center?

4. Were you afraid of medical care staff wearing full personal protective equipment (PPE)?

5. Did you feel anxious about difficulty getting information from outside of the medical center?

6. Did you find it difficult not being able to see your family?

7. Did you contact your family during your hospitalization? (Yes/No)

8. Did you feel that it would have been better to isolate at home while you were being hospitalized?

9. Do you think it is a good idea that asymptomatic patients and patients with mild symptoms are now allowed to isolate at a hotel or at home?

10. Did you feel anxious about life after being discharged?

11. Did you feel anxious if your family, colleagues, and friends would treat you differently after hospitalization?

12. Did you feel anxious if “you might have infected” your family, colleagues, and friends?

13. Did you feel anxious about the possibilities of discrimination and rumors after being discharged?

14. Did you re-evaluate and become more aware of your view on your own health during the hospitalization?

15. Did you regret your infection countermeasures?

16. Did you feel guilty about becoming infected?
